# Gamma-Aminobutyric Acid Production Using Immobilized Glutamate Decarboxylase Followed by Downstream Processing with Cation Exchange Chromatography

**DOI:** 10.3390/ijms14011728

**Published:** 2013-01-15

**Authors:** Seungwoon Lee, Jungoh Ahn, Yeon-Gu Kim, Joon-Ki Jung, Hongweon Lee, Eun Gyo Lee

**Affiliations:** 1 University of Science and Technology (UST), 217 Gajeong-ro, Yuseong-gu, Daejeon 305-350, Korea; E-Mail: swlee@kribb.re.kr; 2 Biotechnology Process Engineering Center, KRIBB, 125 Gwahak-ro Yuseong-gu, Daejeon 305-806, Korea; E-Mails: ahnjo@kribb.re.kr (J.A.); ygkim@kribb.re.kr (Y.-G.K.); jkjung@kribb.re.kr (J.-K.J.); hwlee@kribb.re.kr (H.L.)

**Keywords:** gamma aminobutyric acid, glutamate decarboxylase, glutamic acid, immobilization, cation exchange chromatography

## Abstract

We have developed a gamma-aminobutyric acid (GABA) production technique using his-tag mediated immobilization of *Escherichia coli*-derived glutamate decarboxylase (GAD), an enzyme that catalyzes the conversion of glutamate to GABA. The GAD was obtained at 1.43 g/L from GAD-overexpressed *E. coli* fermentation and consisted of 59.7% monomer, 29.2% dimer and 2.3% tetramer with a 97.6% soluble form of the total GAD. The harvested GAD was immobilized to metal affinity gel with an immobilization yield of 92%. Based on an investigation of specific enzyme activity and reaction characteristics, glutamic acid (GA) was chosen over monosodium glutamate (MSG) as a substrate for immobilized GAD, resulting in conversion of 2.17 M GABA in a 1 L reactor within 100 min. The immobilized enzymes retained 58.1% of their initial activities after ten consecutive uses. By using cation exchange chromatography followed by enzymatic conversion, GABA was separated from the residual substrate and leached GAD. As a consequence, the glutamic acid was mostly removed with no detectable GAD, while 91.2% of GABA was yielded in the purification step.

## 1. Introduction

Gamma-aminobutyric acid (GABA) is a non-proteinaceous amino acid widely used in the food and pharmaceutical industry, where it serves as an inhibitor of neurotransmission with hypotensive and diuretic effects [1–4], as well as in the chemical industry, where it can be employed as a precursor for biodegradable polymers as an intermediate to pyrrolidone to synthesize polymer Nylon 4 [5,6]. The bioconversion of GABA is undergone with glutamate decarboxylase (GAD, EC 4.1.1.15), which catalyzes the GABA conversion from glutamate, while producing CO_2_ [7].

Previous studies on GABA production can be grouped into two categories. As a nutrient source in the food industry, GABA is produced in wild-type *Lactobacillus* strains isolated from various food sources, such as *L. brevis* from Chinese paocai and *L. paracasei* from cheese, yielding several hundred to thousand millimolar GABA with a conversion yield up to 94% in fermentation after optimization [8–14]. Recent advances of fermentative production of GABA using fungal strains, such as *Monascus*, have achieved up to 13.5 g/L of GABA production yield, which resulted in a lower production yield than *Lactobacillus* strains [15–18]. Despite these achievements in microbial fermentation, the enzymatic conversion could be advantageous in terms of a higher conversion yield, GABA concentration and its purity for polymer preparation grade.

Meanwhile, an *Escherichia coli* (*E. coli*) host is frequently used to express recombinant *E. coli* GAD (EGAD) to produce GABA in the biotechnology and pharmaceutical fields [5,6,19–21]. Enzyme immobilization may be additionally employed to recycle GAD for cost efficiency and easy downstream processing. For instance, Lammens *et al.* studied GABA conversion using GAD entrapment in calcium alginate and Eupergit 250 C [5]. Reversible immobilization and reuse of GAD on an affinity support using the cellulose binding domain (CBD) and CBD-tagged GAD was attempted by Park *et al.* [21], showing stable immobilization and reusability of the enzyme. However, the binding capacity of these matrices is limited. Additionally, the GAD purification step may be required prior to GAD immobilization. For this, the use of an immobilized metal affinity chromatography (IMAC) matrix that has high protein binding capacity could integrate immobilization and purification.

In this paper, we have studied enzymatic GABA production from glutamate using immobilized GAD on a metal affinity resin and further purification by cation exchange chromatography. The aim of this process is to convert more than 2 molar concentration of GABA from glutamate and to remove impurities to achieve a purity of higher than 99%, thus potentially providing an alternative method for GABA production for biotechnological and pharmaceutical uses.

## 2. Results and Discussion

### 2.1. GAD Expression and Its Characterization

**Figure 1:**
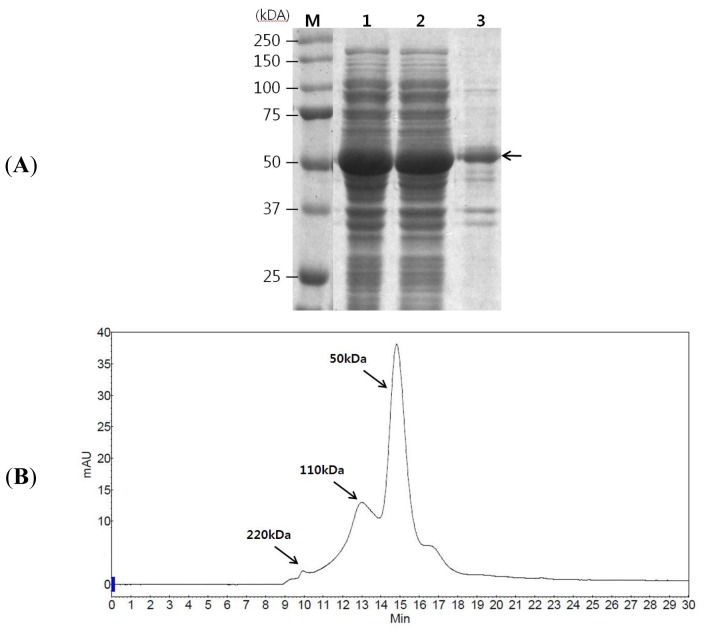
(**A**) Soluble expression of glutamate decarboxylase (GAD). Lane M, molecular (Mw) marker; lane 1, total protein from GAD; lane 2, soluble protein from GAD; lane 3, insoluble protein from GAD; The arrows indicates the position of the expressed GAD. (**B**) GAD formation determined by size exclusion chromatography.

For GAD expression, GAD was fused to six histidine at its *C*-terminus, and the fused gene was placed under the control of a T7 promoter. GAD was expressed in *E. coli* BL21 (DE3) harboring pEKPM-GAD-H6. This expression system can be inhibited by catabolite repression in the presence of excessive glucose, and it is important to minimize the formation of by-products, including acetate in particular, in high-cell-density cultures [22]. Glucose feeding started when the glucose level fell below 0.5 g/L, and this level was thereafter maintained. The culture was maintained for 10 hours, and optical density reached 35 at 600 nm. The target protein EGAD was successfully expressed in a soluble form following induction with 0.4 mM isopropyl-β-D-thio-galactoside (IPTG). About 97.6% of the total GAD expressed was soluble according to SDS-PAGE combined with a densitometer analysis, as shown in Figure 1A, and the purified GAD consisted of 59.7% monomer, 29.2% dimer and 2.3% tetramer (Figure 1B). Previous studies reported that native EGAD mainly forms hexamer [23,24]. The different multimer constitution may be due to conformational distortion from the addition of six histidine to the *C*-terminus. Overall, 2.86 g of soluble EGAD was obtained from the 2 L culture (1.43 g/L). IMAC purification with desalting resulted in 92.1% purity of GAD.

### 2.2. GAD Immobilization and Substrate Selection

**Figure 2:**
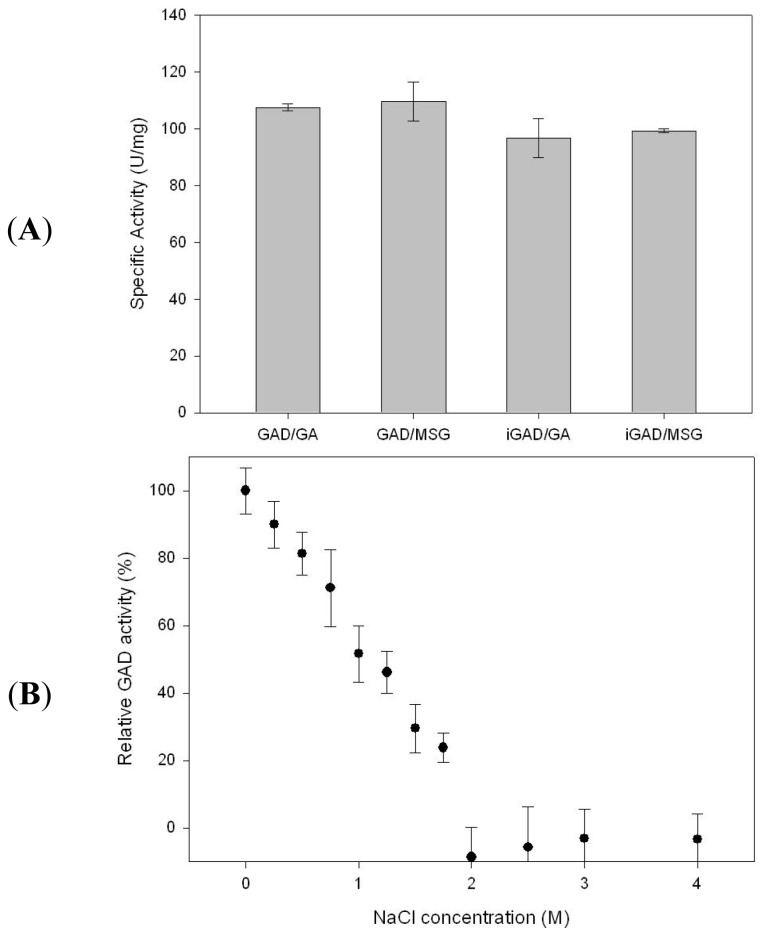
(**A**) Specific activity of free GAD and immobilized GAD (iGAD) with different substrates, glutamic acid (GA) and monosodium glutamate (MSG). (**B**) Effect of NaCl on the specific activity of GAD.

The binding capacity of previously studied matrices was limited to 0.24 and 0.05 mg_GAD_/g_dry bead_ for Eupergit 250 C and calcium alginate, respectively [5], and 1.91 mg_GAD_/g_wet avicel_ for CBD [21]. The advantage of metal affinity resin used in this study is a high binding capacity for enzymes while retaining their activity. When Ni-Sepharose was incubated with GAD at a concentration of 10 mg_GAD_/mL_resin_ for 1 hour, the immobilization yield of GAD on the resin was determined to be 92.3%. There was a slight decrease in specific activity of 10.1% after immobilization (107.6 to 96.7 U/mg), as shown in Figure 2. The decrease in the specific activity may be attributable to his-tag mediated immobilization. Considering that his-tag mediated immobilization is generally effective for maintaining activity of a protein in most cases [25], it is possible that a diffusional limitation generated by the geometry of the matrix could also influence the result.

The specific activity of both free and immobilized GADs towards two different types of substrates, glutamic acid (GA; low solubility in water, less than 60 mM) and monosodium glutamate (MSG; high solubility in water, less than 4.38 M) is compared in Figure 2A. Monosodium glutamate showed higher specific activity than glutamic acid, by 1.8% and 2.7%, for free and immobilized GADs, respectively. Nevertheless, GA is favorable for a high concentration of GABA conversion, where an over-saturated substrate should be used at the reaction pH of 4.0. Since pH increases with substrate conversion due to loss of the carboxyl functional group of glutamate, the acid solution has to be added to avoid GAD activity loss, ultimately resulting in diluted GABA concentration. When over-saturated GA was used, insoluble GA acts as an acid titrate, as it continuously dissolves with soluble glutamate conversion toward GABA and a smaller amount of acidic solution (HCl) is required for titration than MSG. The pH-titration causes accumulation of salt (NaCl from NaOH and HCl), negatively affecting enzymatic activity, as shown in Figure 2B.

### 2.3. GABA Conversion and GAD Recycling

**Figure 3:**
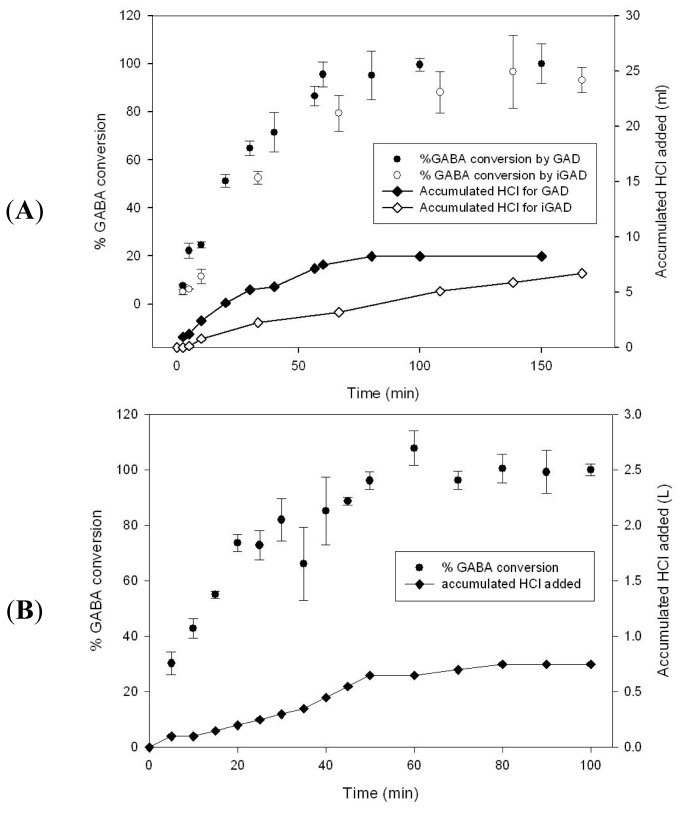
(**A**) 10 mL reactor for conversion of GA to gamma-aminobutyric acid (GABA) by GAD and immobilized GAD (iGAD) (circle) and accumulated HCl added (square). (**B**) 1 L reactor for GABA conversion.

Based on the specific activity performance, as shown in the previous section, GABA conversion by free and immobilized GAD was performed in a reactor with a 10 mL working volume. Figure 3A shows a high concentration of GABA conversion from over-saturated GA. 90% conversion of GABA by immobilized GAD was attained in 100 min and that by free GAD in 60 min, correlating with the difference of specific activity. GABA conversion was terminated at 150 min. To maintain the pH of the reaction solution at 4.0, 1 M HCl was added for free and immobilized GAD conversion, causing product dilution by a factor of 1.82 and 1.74, respectively.

Small-scale conversion was confirmed in a 1 L scale reactor, aiming at more than a 2 M concentration of GABA conversion, as shown in Figure 3B. As a result, 2.17 M GABA was obtained upon termination, while the conductivity was 4.78 mS/cm due to salt formation, and 18 mM GA remained. To our knowledge, the highest GABA concentration was achieved within one hour of processing time. High binding capacity of the Ni-Sepharose matrix allowed concentrated enzyme immobilization and a high rate of conversion. Production of higher concentration GABA may be possible by adding a greater amount of substrate, as long as a low concentration of salt formation is maintained, since increased salt formation could adversely influence the enzyme activity and the subsequent purification step.

**Figure 4:**
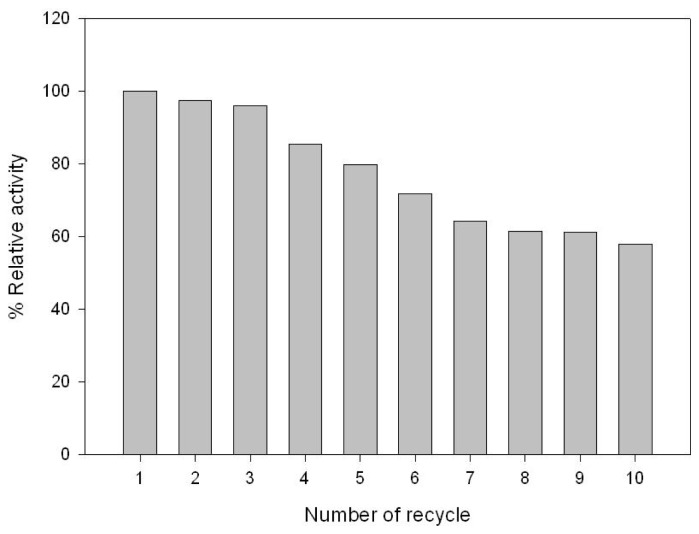
Reusability of immobilized GAD during 10-times recycling.

The immobilized GAD was harvested for reuses. Figure 4 shows the reusability of GAD, indicating that the immobilized GAD retained 58.1% of its initial activity after ten consecutive uses. The loss of GAD activity could be caused by enzyme instability, pH variance and loss of the resin between runs. Although the GAD leakage level is below a quantification limit by BCA method (data not shown), low reaction pH may cause leakage of GAD from the immobilization matrix [25].

### 2.4. GABA Purification by Cation Exchange Chromatography

**Figure 5:**
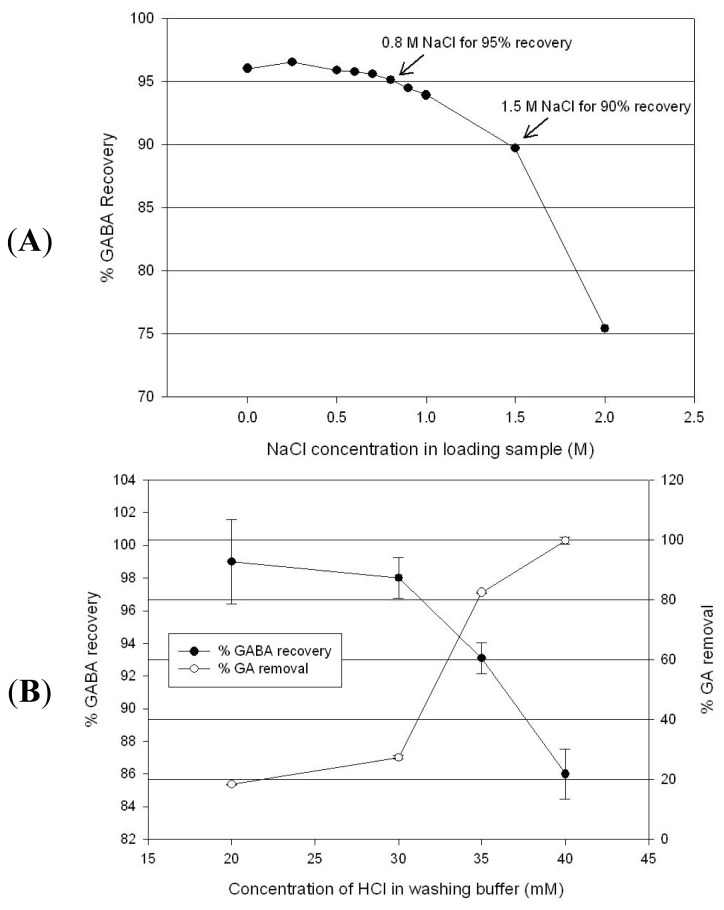
(**A**) NaCl inhibitory effect on GABA binding and (**B**) effect of washing condition on GA removal and GABA recovery.

**Figure 6:**
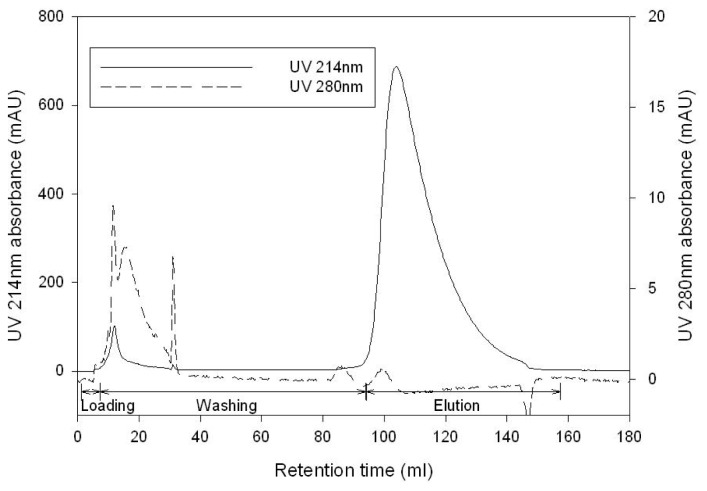
Chromatogram of GABA purification (GABA: 214 nm, GAD: 280 nm).

The purification of GABA was performed using DOWEX 50WX2, a cation exchange resin, to remove the residual substrate and GAD following the enzymatic conversion. In cation exchange chromatographic purification of GABA, the conductivity of the reaction solution and the elution buffer concentration need to be controlled precisely. Figure 5A shows the effect of the salt concentration (NaCl) in the GABA-containing solution. The increased concentration of sodium chloride in the loading sample (reaction solution) negatively affects binding performance, and therefore, sodium chloride has to be maintained below 1 M in order to recover more than 90% of GABA. Figure 5B shows the washing condition used to remove the residual substrate (GA) from GABA. The GABA purity and yield are in a trade-off relation with each other. The optimum washing condition was determined to be 37 mM HCl for 90% GABA retainment and 90% substrate removal. However, the substrate could be separated in a narrow range of 34 to 38 mM HCl due to similar dissociation properties between GABA and GA, and thus, small variation of the buffer B composition in the washing step has a significant impact on both GABA yield and purity. Figure 6 shows the purification performance of GABA solution produced from an enzyme reaction under the optimized chromatography conditions. GABA and GAD were monitored using UV absorbance at 214 and 280 nm, respectively. GABA was eluted at 0.2 M HCl, while GAD flowed through the column in the binding step. As a consequence of cation exchange purification, 18 mM residual substrate (GA) was removed to 0.11 mM (purification factor 12), and GAD in the elution pool was below a detection limit, while 91.2% GABA was yielded.

## 3. Experimental Section

### 3.1. Bacterial Strains and Plasmid Construction

Bacterial strains and plasmid construction for this study have been described by Park *et al.* [21]. *E. coli* DH5α [F^−^, φ80d*lac*ZΔM15, *end*A1 *hsd*R17(rK^−^, mK^+^), *sup*E44, *thi*-1, λ^−^, *rec*A1, *gyr*A96] was used for cloning and plasmid maintenance. *E. coli* BL21 [F^−^, *hsd*S, *gal*, *omp*T, rB^−^, mB^−^] (Novagen, Madison, WI, USA) harboring a lambda derivative, DE3, was used for the expression of GAD. The GAD gene (Genbank: M84025) was amplified from *E. coli* genomic DNA by a polymerase chain reaction (PCR) using a forward primer (5′-GAAGGAGATATACATATGGATAAGAAGCAAGTAACG-3′) and a reverse primer (5′-GTGGTGGTGGTGGTGGTGCTCGAGGGTATGTTTAAAGCT-3′), where the underlined base pairs were introduced for in-fusion ligation. The PCR product was ligated into a modified pET-28b(+) plasmid previously digested with *Bam*HI and *Xho*I using the an In-fusion™ Advantage PCR cloning kit (Clontech Laboratories, Palo Alto, CA, USA), which created pEKPM-GAD-H6.

### 3.2. GAD Expression and Purification

Transformants were cultured overnight in a test tube containing 2 mL of Luria-Bertani (LB) media with kanamycin (50 μg/mL) at 37 °C. The primary seed culture was carried out in a 100 mL baffled flask with a working volume of 20 mL, which was transferred to a 1 L baffled flask containing 200 mL of medium. The medium was composed of (g/L) glucose, 10; yeast extract, 20; soya peptone, 15; MgSO_4_, 1; (NH_4_)_2_SO_4_, 8; NaCl, 0.5; KH_2_PO_4_, 3; Na_2_HPO_4_, 3; and trace elements, supplemented with 50 μg/mL of kanamycin. The seed culture was conducted for 6 hours. The inoculum was transferred to a 5 L fermenter containing 2 L of the same medium as above. When the cell density reached an optical density of 5 at 600 nm, measured by a spectrophotometer (Uvikon 941 plus; Kontron, Zurich, Switzerland), induction was conducted with 0.4 mM IPTG. The cell wall was disrupted by lysozyme-sonication treatment. The cells were incubated for 30 min with lysozyme at 0.2 mg/mL. The sonication was performed at an intensity of 20 with a pulse of 3 seconds for 5 min, which was repeated three times (Ultrasonic Processor; Cole Palmer, Vernon Hills, IL, USA). The cell lysate was centrifuged at 5000 rpm for 10 min, and the supernatant was collected, followed by microfiltration with 0.45 μm pore size.

For the specific activity experiment, GAD was purified by nickel ligand-based his-tag affinity chromatography using HisTrap HP (GE Healthcare, Uppsala, Sweden) and a chromatography system (AKTAexplorer 100Air; GE Healthcare, Uppsala, Sweden) with the following method: 5 column volume (CV) equilibration, sample loading, 10 CV primary washing with buffer A, 10 CV secondary washing by step elution at 5% buffer B, 10 CV step elution at 100% buffer B and re-equilibration. Buffer A was 20 mM sodium phosphate with 500 mM sodium chloride, pH 7.2, and buffer B was 20 mM sodium phosphate with 500 mM sodium chloride and 500 mM imidazole, pH 7.2. The elution fractions were pooled, and their buffer was exchanged with a phosphate saline buffer using Sephadex G-25 (GE Healthcare, Uppsala, Sweden). The purified GAD was then stored at −70 °C for the specific activity measurement.

### 3.3. Immobilization

The purified GAD or GAD-containing cell lysate was immobilized on metal affinity resin, Ni-Sepharose (GE Healthcare, Uppsala, Sweden). GAD was incubated with the resin (1–10 mL) at a concentration of 5–10 mg_GAD_/mL_resin_ for 1 h at room temperature in a binding buffer containing 20 mM sodium phosphate and 500 mM sodium chloride, pH 7.2, and GAD-immobilized resin was centrifuged at 300 rpm for 5 min and washed with the binding buffer three times.

### 3.4. GABA Conversion and GAD Recycling

The GAD reaction conditions used here were the optimized conditions described by Wang *et al.* [26]. For specific activity measurement, 10 μL of the GAD-immobilized resin slurry (consisting of 50% *v*/*v* resin and binding buffer) was transferred to 2 mL screw top microtubes containing 1 mL of reaction buffer; 0.1 M GA or MSG, 0.6 mM CaCl_2_ and 0.15 mM pyridoxal 5′-phosphate pre-incubated at 37 °C and pH 4.0. The enzyme reaction was performed at 37 °C and was terminated after 10 min by heating up to 95 °C. The experiment was performed in triplicates. After reaction, the specific activity was analyzed by measuring the remaining glutamate concentration using a multiparameter bioanalytical system (YSI 7100; YSI Life Science, Yellow Springs, OH, USA). The specific activity measurement was repeated for free (non-immobilized) GAD with the same concentration as employed for immobilized GAD.

GABA conversion was performed in a 100 mL reactor with a working volume of 10 mL, connected to a 718 Stat Titrino (Metrohm AG, Herisau, Switzerland) that can control pH and temperature throughout the reaction. For the GABA conversion experiment, immobilization was carried out without GAD purification, directly from *E. coli* cell lysate, but with the same immobilization procedure as described above. The pH was maintained by titration with 1 M HCl. The solution of 10 mL containing over-saturated 2**–**3 M GA with 0.15 mM pyridoxal 5′-phosphate and 0.6 mM calcium chloride was pre-adjusted to pH 4.0 at 37 °C, and then free or immobilized GAD was added at a concentration of 0.67 mg_GAD_/mL. The pH and temperature were maintained, while the reactor volume was increased with time due to pH titration. Samples of 0.2 mL were collected every 5 to 10 min for measurement of the converted GABA concentration. The conversion was confirmed in a 1 L scale using a 2 L bioreactor with the same procedure as the small scale conversion.

For reusability of immobilized GAD, immobilized GAD at a concentration of 0.67 mg_GAD_/mL was added to 10 mL of a pre-equilibrated solution containing 100 mM GA with 0.15 mM pyridoxal 5′ phosphate and 0.6 mM calcium at pH 4.0 for 30 min. The immobilized GAD was harvested by centrifugation at 300 rpm for 5 min and then washed with a binding buffer for up to ten GAD uses.

### 3.5. Cation Exchange Chromatography

DOWEX 50WX2 (Sigma Aldrich, St. Louis, MO, USA) with a mesh size of 200 was used to purify GABA. The resin (10 mL) was packed in a XK 16/20 column (GE Healthcare, Uppsala, Sweden). Purification was performed with AKTAexplorer with the following method: 5 column volume (CV) equilibration, sample loading, 10 CV primary washing with buffer A, 5 CV secondary washing by step elution at various buffer B concentrations and 5 CV step elution at 100% buffer B. Buffer A and B were deionized water and 0.2 M HCl, respectively. The purification performance was monitored by UV absorbance at 214 nm and 280 nm for GABA and GAD, respectively.

### 3.6. Analysis of GABA, Glutamate and GAD Concentrations

The concentration of GABA was measured by HPLC using a Varian ProStar HPLC system equipped with an Optimapak C18 column (RStech, Daejeon, Korea). The method was carried out with buffer A (tetrahydrofuran/methanol/50 mM sodium acetate pH 6.2, 5:75:420, *v*/*v*/*v*) and buffer B (methanol). A 100 μL sample was mixed with 100 μL sodium bicarbonate, pH 9.8 and a 200 μL dancylchloride solution and incubated at 80 °C for 30 min. After incubation, 8 μL of 10% acetic acid was added prior to HPLC injection. The concentration of glutamate was measured by a multi-parameter bioanalytical system (YSI 7100; YSI Life Science, Yellow Springs, OH, USA). The concentration of protein was measured using a BCA protein assay kit (Thermo Fisher Scientific, Waltham, MA, USA), according to the manufacturer’s instruction.

### 3.7. Characterization of GAD

Soluble GAD expression was analyzed by 10% SDS-PAGE, and quantitative densitometry was performed using a software/scanner system (Uvitec, Cambridge, UK), according to the manufacturer’s instructions.

Multimer formation of GAD was determined by size exclusion HPLC using TSKgel G3000SW (Tosoh Corp., Tokyo, Japan). A purified GAD sample of 20 μL was injected into the column. Isocratic elution was performed at 1 mL/min for 30 min with 0.1 M sodium sulfate in 0.1 M phosphate buffer, pH 6.8, as a mobile phase.

## 4. Conclusions

We have successfully developed a process for GABA production applicable on a large scale. We achieved more than 2 M GABA production in a 1 L scale conversion, and most of the residual substrate and proteins were removed in the downstream part of the process. Active GAD was obtained at 1.43 g/L in *E. coli* and consisted of various multimer formations. GAD was immobilized on the IMAC resin with 92.3% immobilization yield. Based on an investigation of specific enzyme activity and reaction characteristics, we have concluded that GA is preferable as a substrate for the conversion process, resulting in conversion of 2.17 M GABA in a 1 L scale bioreactor. Impurities from the conversion process were removed by cation exchange chromatography under optimized binding and washing conditions. In the future, further investigation is required to achieve a more economically feasible process in terms of an inexpensive stability-enhancing immobilization matrix and engineered GAD obtaining less acidic optimum pH.

## References

[1] Bhargava K.P., Bhattacharya S.S., Srimal R.C. (1964). Central cardiovascular actions of GABA. Br. J. Pharmacol.

[2] Erlander M., Tobin A. (1991). The structural and functional heterogeneity of glutamic acid decarboxylase: A review. Neurochem. Res.

[3] Fenalti G., Law R.H., Buckle A.M., Langendorf C., Tuck K., Rosado C.J., Faux N.G., Mahmood K., Hampe C.S., Banga J.P. (2007). GABA production by glutamic acid decarboxylase is regulated by a dynamic catalytic loop. Nat. Struct. Mol. Biol.

[4] Shelp B.J., Bown A.W., McLean M.D. (1999). Metabolism and functions of gamma-aminobutyric acid. Trends Plant Sci.

[5] Lammens T.M., de Biase D., Franssen M.C.R., Scott E.L., Sanders J.P.M. (2009). The application of glutamic acid α-decarboxylase for the valorization of glutamic acid. Green Chem.

[6] Yamano N., Kawasaki N., Takeda S., Nakayama A. (2012). Production of 2-pyrrolidone from biobased glutamate by using. Escherichia coli. J. Polym. Environ..

[7] MetaCyc http://biocyc.org/META/NEW-IMAGE?type=REACTION&object=GLUTDECARBOX-RXN&redirect=T.

[8] Hiraga K., Ueno Y., Oda K. (2008). Glutamate decarboxylase from *Lactobacillus brevis*: Activation by ammonium sulfate. Biosci. Biotechnol. Biochem.

[9] Komatsuzaki N., Nakamura T., Kimura T., Shima J. (2008). Characterization of glutamate decarboxylase from a high γ-aminobutyric acid (GABA)-producer, *Lactobacillus paracasei*. Biosci. Biotechnol. Biochem.

[10] Komatsuzaki N., Shima J., Kawamoto S., Momose H., Kimura T. (2005). Production of γ-aminobutyric acid (GABA) by *Lactobacillus paracasei* isolated from traditional fermented foods. Food Microbiol.

[11] Li H., Cao Y. (2010). Lactic acid bacterial cell factories for gamma-aminobutyric acid. Amino Acids.

[12] Li H., Cao Y., Cao Y., Xu H. (2008). A high γ-aminobutyric acid-producing *Lactobacillus brevis* isolated from Chinese traditional paocai. Ann. Microbiol.

[13] Li H., Qiu T., Chen Y., Cao Y. (2011). Separation of gamma-aminobutyric acid from fermented broth. J. Ind. Microbiol. Biotechnol.

[14] Ueno Y., Hayakawa K., Takahashi S., Oda K. (1997). Purification and characterisation of glutamate decarboxylase from *Lactobacillus brevis* IFO 12005. Biosci. Biotechnol. Biochem.

[15] Jiang D., Ji H., Ye Y., Hou J. (2011). Studies on screening of higher γ-aminobutyric acid-producing *Monascus* and optimization of fermentative parameters. Eur. Food Res. Technol.

[16] Lee C.L., Pan T.M. (2012). Development of *Monascus* fermentation technology for high hypolipidemic effect. Appl. Microbiol. Biotechnol.

[17] Su Y.C., Wang J.J., Lin T.T., Pan T.M. (2003). Production of the secondary metabolites gamma-aminobutyric acid and monacolin K by *Monascus*. J. Ind. Microbiol. Biotechnol.

[18] Wang J.J., Lee C.L., Pan T.M. (2003). Improvement of monacolin K, gamma-aminobutyric acid and citrinin production ratio as a function of environmental conditions of *Monascus purpureus* NTU 601. J. Ind. Microbiol. Biotechnol.

[19] Le Vo T.D., Kim T.W., Hong S.H. (2012). Effects of glutamate decarboxylase and gamma-aminobutyric acid (GABA) transporter on the bioconversion of GABA in engineered *Escherichia coli*. Bioprocess Biosyst. Eng.

[20] Plokhov A.Y., Gusyatiner M.M., Yampolskaya T.A., Kaluzhsky V.E., Sukhareva B.S., Schulga A.A. (2000). Preparation of γ-aminobutyric acid using *E. coli* cells with high activity of glutamate decarboxylase. Appl. Biochem. Biotechnol.

[21] Park H., Ahn J., Lee J., Lee H., Kim C., Jung J.K., Lee E.G. (2012). Expression, immobilization and enzymatic properties of glutamate decarboxylase fused to a cellulose-binding domain. Int. J. Mol. Sci.

[22] Lee E.G., Baek J.-E., Lee S.-H., Kim T.-W., Choi J.H., Rho M.-C., Ahn J.-O., Lee H.-W., Jung J.-K. (2009). Efficient proteolytic cleavage by insertion of oligopeptide linkers and its application to production of recombinant human interleukin-6 in *Escherichia coli*. Enzyme Microb. Technol.

[23] Capitani G., de Biase D., Aurizi C., Gut H., Bossa F., Grutter M.G. (2003). Crystal structure and functional analysis of *Escherichia coli* glutamate decarboxylase. EMBO J.

[24] Fonda M.L. (1985). L-Glutamate decarboxylase from bacteria. Meth. Enzymol.

[25] Nakanishi K., Sakiyama T., Kumada Y., Imamura K., Imanaka H. (2008). Recent advances in controlled immobilization of proteins onto the surface of the solid substrate and its possible application to proteomics. Curr. Proteomics.

[26] Wang Q., Xin Y., Zhang F., Feng Z., Fu J., Luo L., Yin Z. (2011). Enhanced γ-aminobutyric acid-forming activity of recombinant glutamate decarboxylase (gadA) from *Escherichia coli*. World J. Microbiol. Biotechnol.

